# Growth, photosynthesis, and nutrient uptake in wheat are affected by differences in nitrogen levels and forms and potassium supply

**DOI:** 10.1038/s41598-018-37838-3

**Published:** 2019-02-04

**Authors:** Jiuxin Guo, Yamin Jia, Huanhuan Chen, Lijun Zhang, Jinchang Yang, Jun Zhang, Xiangyu Hu, Xin Ye, Yan Li, Yi Zhou

**Affiliations:** 1 International Magnesium Institute, College of Resources and Environment, Fujian Agriculture and Forestry University, Fuzhou, 350002 China; 2 Institute of Plant Nutritional Physiology and Molecular Biology, College of Resources and Environment, Fujian Agriculture and Forestry University, Fuzhou, 350002 China; 3 The Rice Research Institute of Guangdong Academy of Agricultural Sciences, Guangzhou, 510640 China; 4 Key Laboratory of Bio-organic Fertilizer Creation, Ministry of Agriculture, College of Resource and Environment, Anhui Science and Technology University, Bengbu, 233100 China

## Abstract

Nitrogen (N) and potassium (K) are essential macronutrients for plants growth; however, the mechanism by which K mediates negative effects on ammonium-sensitive plants is still poorly understood. We hypothesized that K supplies may enhance antagonistic ammonium stress while improving nitrate nutrition function, which wheat seedlings were grown in sand culture in the presence of two N forms (ammonium; nitrate) supplied at two rates (2, 10 mmol L^−1^) and three K levels (0.5, 5, 15 mmol L^−1^). We found that a high N rate increased plant biomass under nitrate nutrition, while it had a negative effect under ammonium nutrition. Compared with nitrate, biomass was depressed by 54% or 85% for low or high N rate under ammonium. This resulted in a reduction in gas exchange parameters and a subsequent decrease in growth variables and nutrient uptake, whereas these parameters increased significantly with increasing K levels. Moreover, in principal components analysis, these variations were highly clustered under nitrate nutrition and highly separated under ammonium nutrition. Our study shows a clear positive interaction between K and N, suggesting that high K supply relieves ammonium stress while improving growth vigor under nitrate nutrition by enhancing nutrient uptake and assimilate production in wheat plants.

## Introduction

Nitrogen (N) is a key element required for plant growth, and is one of the most important yield-limiting nutrients in crop production in all agro-ecological regions of the world. N is commonly taken up from the soil in one of two inorganic forms: ammonium (NH_4_^+^) and nitrate (NO_3_^−^)^1–3^. Different N forms can affect the physiological and metabolic processes of plants, such as nutrient uptake, enzyme activity, photosynthesis rate, respiration rate, water balance, and signaling pathways, thus eventually influencing plant growth and crop yield^3–6^. Although NH_4_^+^ is an intermediate in many metabolic reactions, it can result in toxicity symptoms in many higher plants when supplied as the sole N source^1,7–9^.

Sole NH_4_^+^ supply provokes negative effects on NH_4_^+^-sensitive plants. The effects of NH_4_^+^ and NO_3_^−^ nutrition on plant growth have been intensively studied; however, the results are not consistent and depend mainly on plant species. Wheat and maize^10^, sugar beet^11^, beans^12–15^, tobacco^16,17^ and canola^18^, grow preferentially on NO_3_^−^ nutrition; whereas, rice^19–21^, pine and larch^22^, grow preferentially on NH_4_^+^ nutrition. The mechanisms responsible for NH_4_^+^ toxicity have been the subject of much speculation, and proposals include proton extrusion associated with NH_4_^+^ uptake, cytosolic pH disturbances, decreased water use efficiency, shifts in plant carbohydrate status, the uncoupling of photophosphorylation, high energy costs of membrane transport, and displacement of crucial cations, such as K^4,7,9,12–14^.

NH_4_^+^ nutrition has been found to cause strong inhibition of potassium (K) uptake by plants^4,16,17^. K, which is an essential nutrient involved in many important plant physiological processes, can improve crop yield and quality and enhance stress tolerance^23,24^. Thus, N and K requirements and management of these essential nutrients for crop production have become a focus of research into the interactions between N and K in terms of factors such as form and rate. Currently, the imbalanced fertilizer use is common in field production in many developing countries. The practice often leads to an excess of soil N combined with a serious and continual depletion of soil K, mainly due to the application of excessive N and inadequate K^25^. Over-application of N is a serious problem in intensive agricultural production areas because this leads to enrichment of reactive N constituents in the environmental, soil acidification and also affects the transformation of soil N forms, with consequent impairment of ecosystems^26–28^. It is generally accepted that, regardless of the application of N fertilizer or not, the progress of N form transformation is inhibited by the interaction between NH_4_^+^ and NO_3_^−^, with higher NH_4_^+^ and lower NO_3_^−^ status maintained in acidic soil^29,30^ and low oxygen or waterlogged conditions^31,32^.

Wheat is one of the most important cereal crops worldwide and grows preferentially under NO_3_^−^ nutrition; however, its production is challenged by waterlogging of N forms, which is reported to cause yield losses between 15 and 20%^33^, and frequently occurs in regions with heavy rainfall and high ground water levels. Waterlogging causes significant reduction in gas diffusion and thereby, creates high NH_4_^+^ conditions in soil^31,32^, leading to a substantial decrease in wheat growth and crop yield^33^. Most studies have shown that the application of NH_4_^+^ as the sole N source decreased biomass in wheat compared with that achieved under NO_3_^−^ nutrition^10,34,35^. In addition, changes in NH_4_^+^/NO_3_^−^ ratios and K supply levels under soil culture conditions influence the yield and nutrient uptake of wheat plants^36^. Thus, we hypothesized that plant growth and crop yield are influenced not only by the amount of available N in the soil, but also by the N forms, and that the process can be regulated by K supply.

Although the individual effects of different N forms on plant growth have been widely studied, the combined effects of various levels of N forms and K supply on wheat growth and crop yield are largely unknown. Similarly, little is known about the effects of K supply relative to N forms on the photosynthetic process and nutrient uptake in wheat plants. In this study, we investigated the effects of different levels of N forms and K supply on growth in wheat plants, primarily by investigating biomass, growth, gas exchange, and N and K uptake.

## Results

### Wheat growth

**Figure 1:**
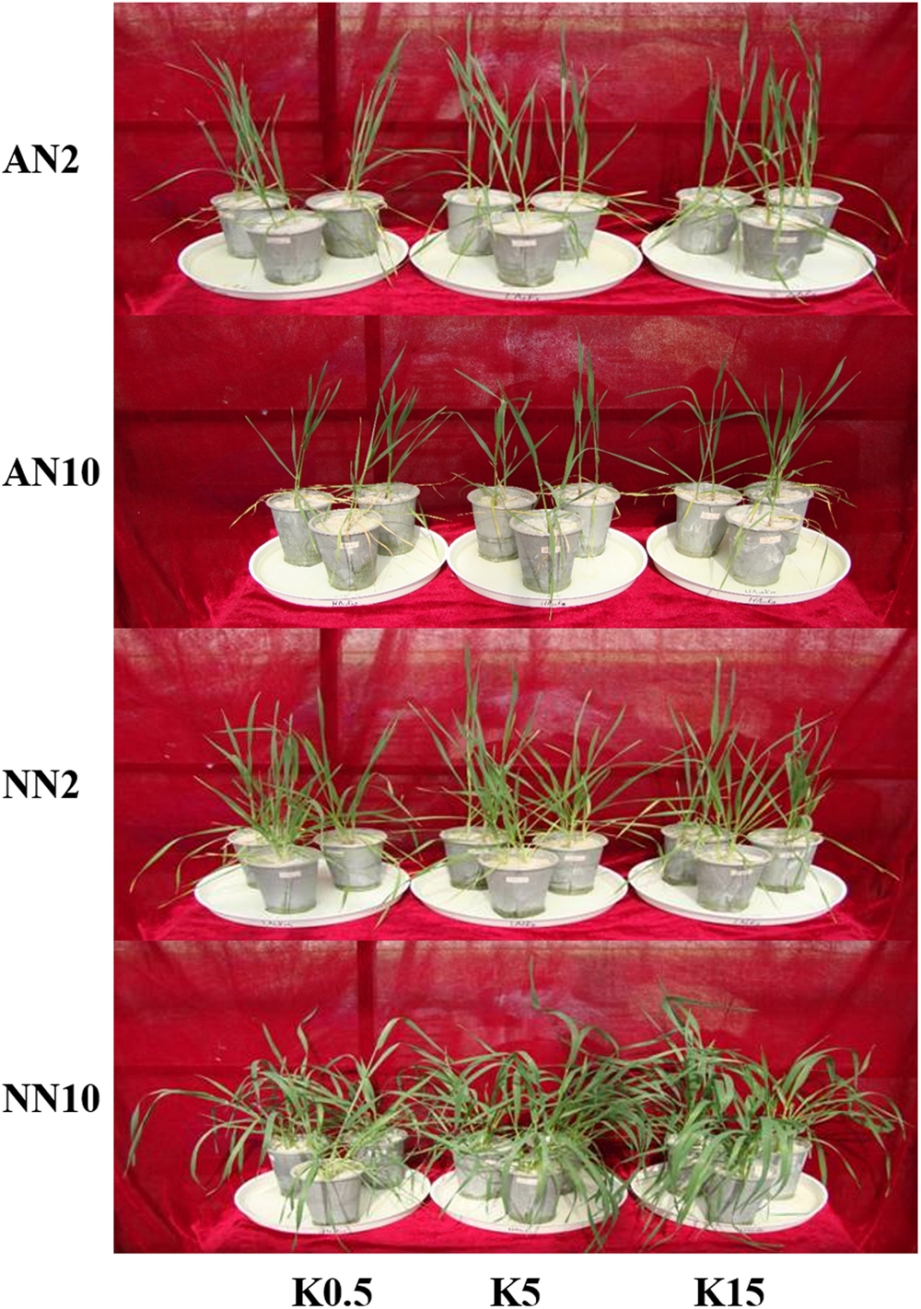
Effects of different levels of N forms and K supply on the plant growth in wheat seedlings. Wheat plants were supplied with ammonia (AN) and nitrate (NN) at 2 and 10 mmol L^−1^ and three different concentrations of potassium (K) solution (0.5, 5, 15 mmol L^−1^ K [K0.5, K5, K15]).

Significant differences in plant biomass were observed between those supplied with NH_4_^+^ and NO_3_^−^ forms of N at two rates (Table 1). N supplied at the high rate in the form of NO_3_^−^ significantly increased the biomass of different organs. In contrast, NH_4_^+^ resulted in a reduction in plant biomass that was exacerbated by high NH_4_^+^. However, the plant biomass increased with K levels under both NH_4_^+^ and NO_3_^−^ nutrition. Compared with NO_3_^−^ supply in the absence of K supply, the biomass of root, stem, leaf, and panicle in plants fed NH_4_^+^ was reduced by 67%, 54%, 53% and 40% at the low N rate and by 87%, 88%, 88% and 77% at the high N rate, respectively. These results were also clearly supported by the images collected during the culture stage (Fig. 1). Regardless of the N form, the high N rate significantly decreased the root:shoot ratios. In contrast to the effects of NH_4_^+^, the root:shoot ratios of plants supplied NO_3_^−^ increased with K levels, and the highest ratio was observed under low NO_3_^−^ treatment. A clear positive interaction was observed among N forms and rates and K levels on the biomass of the different organs except the panicle; however, there were no significant differences in the root:shoot ratios among the different K levels.

**Table 1:** Effects of different levels of N forms and K supply on plant dry weight and root:shoot ratios in wheat plants.

Treatments		Root (g plant^−1^)	Stem (g plant^−1^)	Leaf (g plant^−1^)	Panicle (g plant^−1^)	Root:shoot
AN2	K0.5	0.132 ± 0.009 g	0.230 ± 0.011 fg	0.126 ± 0.006 g	0.433 ± 0.021 i	0.168 ± 0.012 d
	K5	0.145 ± 0.009 fg	0.252 ± 0.007 f	0.136 ± 0.006 fg	0.519 ± 0.026 h	0.160 ± 0.008 de
	K15	0.153 ± 0.005 f	0.280 ± 0.009 f	0.167 ± 0.008 f	0.572 ± 0.015 g	0.151 ± 0.009 e
AN10	K0.5	0.070 ± 0.001 h	0.162 ± 0.009 h	0.112 ± 0.007 g	0.255 ± 0.008 k	0.133 ± 0.006 f
	K5	0.085 ± 0.001 h	0.174 ± 0.003 h	0.126 ± 0.006 g	0.364 ± 0.009 j	0.128 ± 0.001 f
	K15	0.086 ± 0.002 h	0.181 ± 0.008 gh	0.135 ± 0.006 fg	0.375 ± 0.005 j	0.124 ± 0.001 f
NN2	K0.5	0.364 ± 0.008 e	0.514 ± 0.054 e	0.269 ± 0.015 e	0.777 ± 0.040 f	0.234 ± 0.015 c
	K5	0.432 ± 0.017 d	0.552 ± 0.003 de	0.310 ± 0.002 d	0.856 ± 0.024 e	0.251 ± 0.007 b
	K15	0.491 ± 0.012 c	0.600 ± 0.003 d	0.341 ± 0.007 d	0.903 ± 0.009 d	0.266 ± 0.006 a
NN10	K0.5	0.500 ± 0.013 c	1.243 ± 0.067 c	0.688 ± 0.082 c	1.339 ± 0.038 c	0.153 ± 0.004 e
	K5	0.639 ± 0.012 b	1.521 ± 0.059 b	1.065 ± 0.066 b	1.411 ± 0.009 b	0.160 ± 0.002 de
	K15	0.742 ± 0.024 a	1.681 ± 0.031 a	1.300 ± 0.050 a	1.485 ± 0.043 a	0.166 ± 0.010 d
**Significance (** ***P*** **)**						
N form (NF)		<0.001	<0.001	<0.001	<0.001	<0.001
N rate (NR)		<0.001	<0.001	<0.001	<0.001	<0.001
K level (K)		<0.001	<0.001	<0.001	<0.001	NS
NF*NR		<0.001	<0.001	<0.001	<0.001	<0.001
NF*K		<0.001	<0.001	<0.001	NS	<0.001
NR*K		<0.001	<0.001	<0.001	NS	NS
NF*NR*K		<0.001	<0.001	<0.001	NS	NS

Data presented as mean ± standard deviation of three replicates (Mean ± SD, n = 3). Different lowercase letters in the same column indicate significant differences among treatments at *P* < 0.05 level.

In accordance with the effects on biomass, the other growth variables (root volume, number of tillers, flag leaf area, total leaf area and specific leaf weight) also changed with the N forms and rates in the presence of K, with the exception of the number of tillers at different K levels and specific leaf weight under treatment with the different N forms (Table 2). Overall, NO_3_^−^ dramatically increased the growth variables, with greater increases under high NO_3_^−^ treatment than those under low NO_3_^−^ treatment. In contrast to the effects of NO_3_^−^ alone, the high N rate had a negative effect on growth variables in plants fed NH_4_^+^, whereas no significant differences in root volume, number of tillers and specific leaf weight were observed between the N rates.

**Table 2:** Effects of different levels of N forms and K supply on growth variables (root volume, number of tillers, flag leaf area, total leaf area and specific leaf weight) in wheat plants.

Treatments		Root volume (cm^3^ plant^−1^)	Number of tillers (No. plant^−1^)	Flag leaf area (cm^2^ leaf^−1^)	Total leaf area (cm^2^ plant^−1^)	Specific leaf weight (g m^−2^)
AN2	K0.5	1.40 ± 0.04 d	1.00 ± 0.00 d	8.07 ± 0.64 h	32.49 ± 0.88 hi	38.64 ± 1.13 c
	K5	1.62 ± 0.06 d	1.17 ± 0.29 d	10.25 ± 0.66 fg	38.02 ± 0.74 h	35.67 ± 0.99 de
	K15	1.85 ± 0.09 d	1.33 ± 0.29 d	10.92 ± 0.45 f	50.27 ± 1.50 g	33.17 ± 0.73 f
AN10	K0.5	0.92 ± 0.02 d	1.00 ± 0.00 d	6.09 ± 0.58 i	27.46 ± 1.86 i	40.89 ± 1.28 b
	K5	1.03 ± 0.05 d	1.00 ± 0.00 d	8.22 ± 0.41 h	34.39 ± 1.08 hi	36.73 ± 0.72 d
	K15	1.15 ± 0.09 d	1.00 ± 0.00 d	9.63 ± 0.53 g	40.80 ± 0.82 h	33.15 ± 1.81 f
NN2	K0.5	3.65 ± 0.24 c	2.00 ± 0.50 c	10.77 ± 0.13 f	77.29 ± 0.85 f	34.75 ± 1.71 ef
	K5	4.27 ± 0.16 c	2.17 ± 0.29 c	12.15 ± 0.49 e	91.87 ± 1.05 e	33.78 ± 0.61 f
	K15	4.95 ± 0.28 c	2.33 ± 0.29 c	13.65 ± 0.17 d	125.72 ± 1.25 d	27.14 ± 0.33 g
NN10	K0.5	5.33 ± 0.15 b	3.50 ± 0.50 b	18.08 ± 1.37 c	178.64 ± 10.67 c	42.94 ± 0.64 a
	K5	5.90 ± 0.09 ab	3.83 ± 0.29 ab	23.66 ± 0.77 b	264.92 ± 11.14 b	41.47 ± 0.35 ab
	K15	6.26 ± 0.09 a	4.00 ± 0.00 a	26.21 ± 0.83 a	336.29 ± 11.30 a	38.65 ± 1.31 c
**Significance (** ***P*** **)**						
N form (NF)		<0.001	<0.001	<0.001	<0.001	NS
N rate (NR)		<0.001	<0.001	<0.001	<0.001	<0.001
K level (K)		<0.001	NS	<0.001	<0.001	<0.001
NF*NR		<0.001	<0.001	<0.001	<0.001	<0.001
NF*K		<0.001	NS	<0.01	<0.001	<0.05
NR*K		NS	NS	<0.001	<0.001	NS
NF*NR*K		NS	NS	<0.001	<0.001	<0.01

Data presented as mean ± standard deviation of three replicates (Mean ± SD, n = 3). Different lowercase letters in the same column indicate significant differences among treatments at *P* < 0.05 level.

### Gas exchange and relative chlorophyll content

There were significant differences in gas exchange parameters (*P*_n_, *g*_s_, *C*_i_, and *T*_r_) and relative chlorophyll content (SPAD value) between plants under NH_4_^+^ and NO_3_^−^ nutrition at different N rates and K supply conditions (Table 3). The *P*_n_, *g*_s_, and *T*_r_ values increased significantly with the K levels, while *C*_i_ decreased. However, under high NH_4_^+^ nutrition, the high N rate restrained *P*_n_, *g*_s_, and *T*_r_ by 6%, 12% and 7%, respectively, while position effects were observed under NO_3_^−^ nutrition. Under NO_3_^−^ nutrition, the SPAD of flag leaves was decreased by 11% under low N rate compared with that under the high N rate, while no significant differences in SPAD were observed between plants under NH_4_^+^ nutrition and K levels.

**Table 3:** Effects of different levels of N forms and K supply on leaf photosynthetic rate (*P*_n_), stomatal conductance (*g*_s_), intercellular CO_2_ concentration (*C*_i_), transpiration rate (*T*_r_), and relative chlorophyll content (SPAD value, n = 6) in wheat plants.

Treatments		*P*_n_ (μmol m^−2^ s^−1^)	*g*_s_ (mol H_2_O m^−2^ s^−1^)	*C*_i_ (μmol CO_2_ mol^−1^)	*T*_r_ (mmol H_2_O m^−2^ s^−1^)	Relative chlorophyll content (SPAD)
AN2	K0.5	16.96 ± 0.23 fgh	0.267 ± 0.005 cd	298 ± 1 b	4.80 ± 0.18 g	50.83 ± 2.51 a
	K5	17.40 ± 0.23 ef	0.282 ± 0.005 c	290 ± 5 bcd	5.02 ± 0.06 fg	50.32 ± 1.29 a
	K15	18.01 ± 0.21 de	0.288 ± 0.010 bc	281 ± 4 de	5.45 ± 0.16 de	49.80 ± 2.07 a
AN10	K0.5	16.03 ± 0.59 h	0.233 ± 0.014 e	319 ± 3 a	4.44 ± 0.22 h	50.42 ± 1.41 a
	K5	16.30 ± 0.17 gh	0.249 ± 0.006 de	312 ± 10 a	4.74 ± 0.31 gh	50.67 ± 3.23 a
	K15	16.75 ± 0.55 fgh	0.255 ± 0.008 de	296 ± 1 bc	5.07 ± 0.06 fg	49.58 ± 4.48 a
NN2	K0.5	17.02 ± 0.54 fg	0.307 ± 0.017 b	288 ± 6 cd	5.28 ± 0.30 ef	45.95 ± 1.91 b
	K5	18.56 ± 0.55 cd	0.336 ± 0.024 a	281 ± 6 de	6.05 ± 0.30 c	44.32 ± 1.15 b
	K15	19.29 ± 0.80 bc	0.347 ± 0.015 a	275 ± 4 ef	6.51 ± 0.19 b	44.27 ± 2.68 b
NN10	K0.5	18.75 ± 0.68 cd	0.333 ± 0.029 a	272 ± 5 f	5.72 ± 0.09 cd	51.06 ± 2.18 a
	K5	19.74 ± 0.82 ab	0.342 ± 0.010 a	267 ± 7 fg	6.53 ± 0.19 b	50.10 ± 1.55 a
	K15	20.30 ± 0.91 a	0.352 ± 0.012 a	259 ± 6 g	7.13 ± 0.13 a	49.75 ± 1.60 a
**Significance (** ***P*** **)**						
N form (NF)		<0.001	<0.001	<0.001	<0.001	<0.001
N rate (NR)		NS	<0.05	NS	NS	<0.001
K level (K)		<0.001	<0.001	<0.001	<0.001	NS
NF*NR		<0.001	<0.001	<0.001	<0.001	<0.001
NF*K		NS	NS	NS	<0.001	NS
NR*K		NS	NS	NS	NS	NS
NF*NR*K		NS	NS	NS	NS	NS

Data presented as mean ± standard deviation of three replicates (Mean ± SD, n = 3). Different lowercase letters in the same column indicate significant differences among treatments at *P* < 0.05 level.

### N and K content

**Figure 2:**
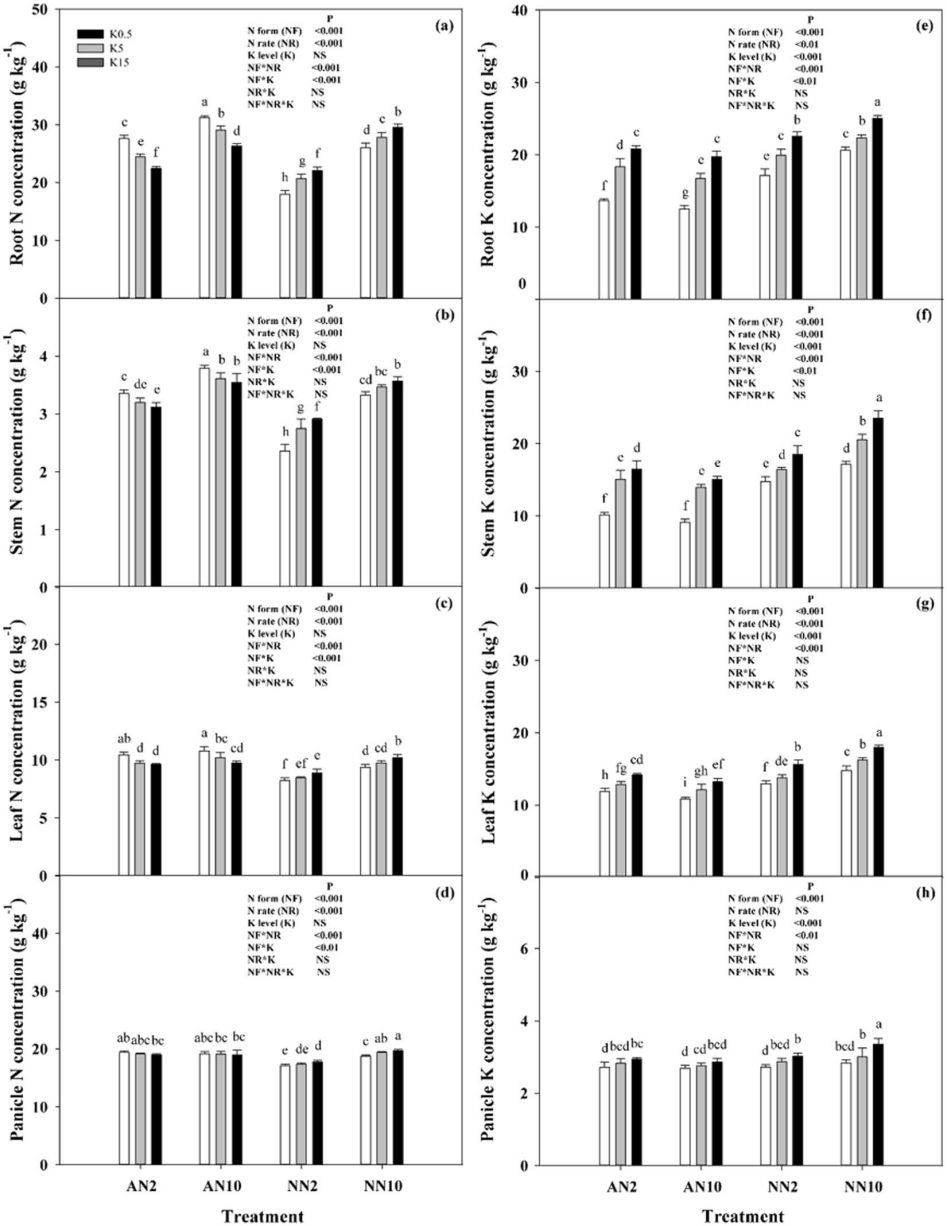
Effects of different levels of N forms and K supply on the N (**a**–**d**) and K (**e**–**h**) concentrations in wheat roots, stems, leaves, and panicles. Wheat plants were supplied with ammonia (AN) and nitrate (NN) at 2 and 10 mmol L^−1^ and three different concentrations of potassium (K) solution (0.5, 5, 15 mmol L^−1^ K [K0.5, K5, K15]). Data represent the mean ± standard deviation of three replicates. Significant differences (*P* < 0.05) were determined by different lowercase letters according to a one-way or multi-way ANOVA followed by Duncan’s multiple range test. NS, no significant difference.

**Figure 3:**
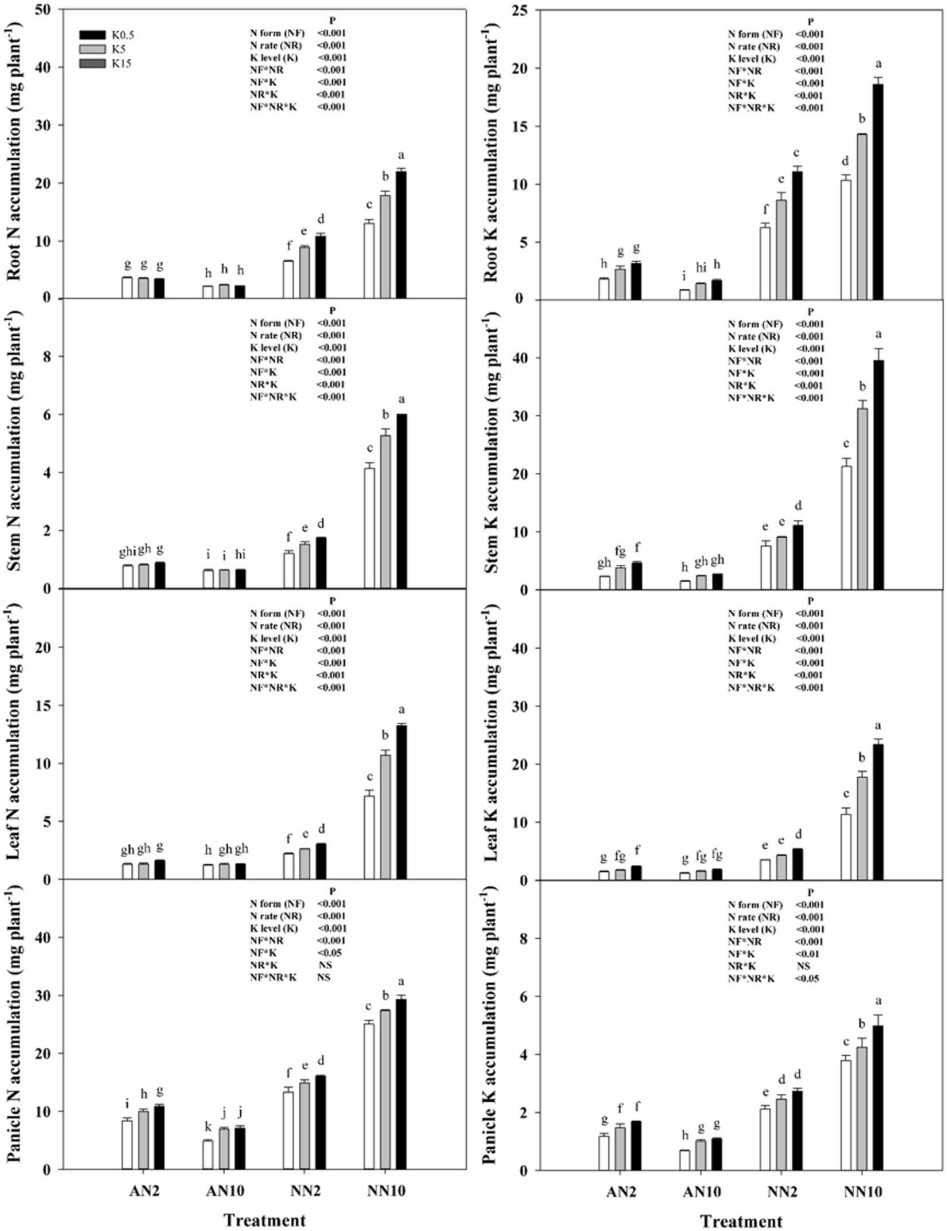
Effects of different levels of N forms and K supply on the N (**a**–**d**) and K (**e**–**h**) accumulations in wheat roots, stems, leaves, and panicles. Wheat plants were supplied with ammonia (AN) and nitrate (NN) at 2 and 10 mmol L^−1^ and three different concentrations of potassium (K) solution (0.5, 5, 15 mmol L^−1^ K [K0.5, K5, K15]). Data represent the mean ± standard deviation of three replicates. Significant differences (*P* < 0.05) were determined by different lowercase letters according to a one-way or multi-way ANOVA followed by Duncan’s multiple range test. NS, no significant difference.

The different N forms and rates supplied with K had a significant effect on both N and K concentration and accumulation in wheat at the harvest period (Figs 2 and 3). Independent of K levels, the N concentration of different organs (including root, stem, leaf, and panicle) were regulated by both N form and rate (Fig. 2a–d), with generally higher concentrations under a high N rate than under a low N rate. Furthermore, the organ N concentrations were higher under NH_4_^+^ nutrition than under NO_3_^−^ nutrition. Moreover, in contrast to the results of NO_3_^−^, the organ N concentrations decreased with increasing K levels under NH_4_^+^ nutrition. On the other hand, under NO_3_^−^ nutrition, the organ K concentrations under the high N rate were higher than those under the low N rate, while a negative effect of N rate on organ K concentrations was observed under NH_4_^+^ nutrition. Regardless of N forms and rates, the organ K concentrations increased with K levels (Fig. 2e–h).

The accumulation of N and K was similar to pattern of changes in the K concentration (Fig. 3). Overall, compared with the low N rate, the average N accumulation in root, stem, leaf and panicle was decreased by 35%, 48%, 23% and 38%, respectively, by the high N rate under NH_4_^+^ nutrition and without K. The average K accumulation was also decreased by 10%, 19%, 35% and 36%, respectively. However, the accumulation of both N and K in organs was increased by the high N rate under NO_3_^−^ nutrition.

**Figure 4:**
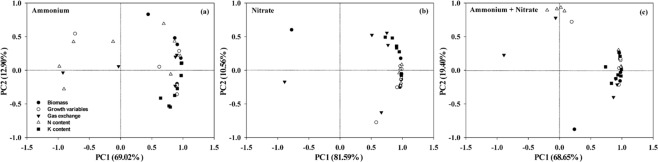
Principal component analysis (PCA) of ammonium (**a**), nitrate (**b**), and ammonium plus nitrate (**c**) based on growth and physiological parameters under different N rate with K supply treatments.

The effects of the variations in NH_4_^+^, NO_3_^−^ and NH_4_^+^ plus NO_3_^−^ among the different N rate with K supply treatments were further evaluated by PCA (Fig. 4). The results showed that the growth and physiological parameters were significantly separated under the different N forms, with the first two principal components accounting for 81.92% (69.02% for PC1 and 12.90% for PC2), 92.15% (81.59% for PC1 and 10.56% for PC2) and 88.05% (68.65% for PC1 and 19.40% for PC2) of the total variations under NH_4_^+^, NO_3_^−^ and NH_4_^+^ plus NO_3_^−^, respectively. According to the PCA, the total variations were highly clustered under NO_3_^−^ nutrition. In contrast, the variations were less clustered under NH_4_^+^ nutrition, especially the N content and gas exchange parameters.

### K^+^ uptake rate

**Figure 5:**
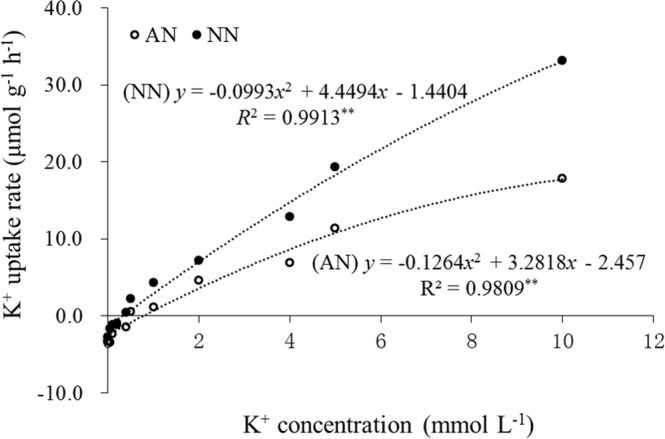
Effects of N forms on K^+^ uptake by the roots of wheat at the seedling stage. Wheat plants were supplied with ammonia (AN) and nitrate (NN) at 2 mmol L^−1^. Data represent the mean of five replications.

As shown in Fig. 5, there was a significant difference in K^+^ uptake under the two N forms. Compared with NH_4_^+^ nutrition, the K^+^ uptake of wheat seedlings was increased by different K^+^ concentration under NO_3_^−^ nutrition, while the K^+^ uptake rate under NO_3_^−^ nutrition (4.4494) was increased by 36% compared with that under NH_4_^+^ nutrition (3.2818) based on regression analysis.

## Discussion

Unlike plants such as rice^19,21^ and pine^22^, wheat is a typical low NH_4_^+^ tolerance plant, with toxicity symptoms manifested as a decline in growth and yield^1,2,7^. Generally speaking, wheat is not always confronted with a soil environment of high NH_4_^+^, except under special circumstances, such as heavy rainfall or irrigation and poor soil drainage combined with excess N fertilization^31,32^, which is especially common in wheat planting regions in the middle and lower of the Yangtze River Plain in southern China^37^.

### N forms and rates affecting growth and photosynthetic CO_2_ fixation in wheat

Plant growth is clearly affected by the forms of N supplied as nutrient. In the present study, we showed that the biomass of both non-reproductive and reproductive organs was depressed by NH_4_^+^ supply, and resulted in lower root:shoot ratios, indicating the negative effects of pure NH_4_^+^ nutrition on wheat growth (Table 1). In accordance with this, Huang *et al*.^35^ reported that root and shoot biomass of wheat plants was lower when cultured in solution with NH_4_^+^ as a N source than that observed in the presence of NO_3_^−^. Furthermore, Wang *et al*.^38^ demonstrated that wheat yield was higher when using NO_3_^−^ fertilizer than that achieved using NH_4_^+^ fertilizer under field conditions. Some studies showed that NH_4_^+^ nutrition resulted in differences in shoot:root ratios compared with those obtained under NO_3_^−^ nutrition in wheat^10^, bean^12,14,15^ and canola^18^. These results were further confirmed in other studies of rice varieties^5,19,20,39,40^, which were found to be tolerant to pure NH_4_^+^ nutrition. In contrast to these findings, Walch-Liu *et al*.^41^ reported that the root:shoot ratios was higher under NH_4_^+^-fed than that under NO_3_^−^-fed in two tobacco cultivars. Moreover, the effect of N forms on root:shoot ratios is mediated by regulation of the N rate. Lu *et al*.^17^ identified a more marked positive relationship between root:shoot ratios under low (2 mmol L^−1^) or high (15 mmol L^−1^) NH_4_^+^ supply compared with those obtained under NO_3_^−^ supply in tobacco plants. Zhou *et al*.^42^ also observed that higher root:shoot ratios were obtained with higher NH_4_^+^ supply (5 mmol L^−1^) than that using NO_3_^−^ as a N supply, although lower N supply (1 mmol L^−1^) had no effect on the ratios in cucumber plants. Similar results were obtained in the present study, with consistently lower root:shoot ratios using NH_4_^+^ than those with NO_3_^−^, while high N reduced the ratio regardless of the N form (Table 1). However, dynamic changes in root:shoot ratios at various sampling points have also been observed in wheat^35^ and sugar beet^11^. These results indicate that the effects of N forms on root and shoot growth in different species and at different N rates and sample points are mediated primarily by altering the partitioning of fixed carbon (C) during photosynthesis in both root and shoot.

These results was consistent with those reported by Guo *et al*.^3^ and Gao *et al*.^18^, who suggested that the conflicting effects of N forms on plant growth are related to the gas exchange parameter *P*_n_, which is associated with *g*_s_ and also *C*_i_. The lower biomass of different organs in wheat plants under NH_4_^+^ nutrition compared to those under NO_3_^−^nutrition was consistent with the observed differences in the gas exchange parameters (Table 3). Thus, it can be concluded that the reduced carbon assimilation in NH_4_^+^-fed wheat plants may have been important in contributing to reduced growth. The higher *P*_n_ and higher root:shoot ratios of the wheat plants under NO_3_^−^nutrition ensure greater availability and allocation of carbon to the root than to the shoot, thus improving plant growth. In addition, the inhibition of wheat growth in response to NH_4_^+^ application could be attributed mainly to a reduction in growth variables including root volume, number of tillers, and leaf area (flag leaf and total leaf), with the response intensified under high N conditions (Table 2). Similar results have been reported for sugar beet^11^ and tobacco^41^, in which the negative effect of NH_4_^+^ nutrition on leaf growth was explained by either increased chlorophyll content and chloroplast number and volume, reduced cell number and cell expansion, or by osmotic regulation^43^ and water uptake in bean plants^12–14^. However, differences in biomass production, gas exchange, root and leaf morphological and physiology observed under conditions of different N forms^5,6,21,40,41,44^, indicate that the N forms available affect plant growth and photosynthesis.

### N forms and rates affecting nutrient uptake in wheat

N form and rate affect plant growth by regulating photosynthetic carbon fixation and distribution as well as plant nutrient uptake. Plant N concentrations in different organs (root, stem, leaves and panicle) were increased in response to NH_4_^+^ application with two N rates (Fig. 2). Brück and Guo^15^ reported that N concentrations in young, fully developed leaves under NH_4_^+^-fed were significantly increased by 59% compared to leaves under NO_3_^−^-fed in beans, and reduced in wheat, maize^10^ and rice^19^. Thus, plant N concentrations under NH_4_^+^ nutrition were consistently higher under NO_3_^−^ nutrition, whether it is a prefer ammonium plant or a prefer nitrate plant, indicating that the growth of NH_4_^+^-fed plants was not limited by N availability. In PCA results, the growth and physiological parameters were significantly separated by the different N forms, with higher levels of variation clusters under NO_3_^−^ nutrition than those under NH_4_^+^ nutrition, especially in terms of N content and gas exchange parameters (Fig. 4). These findings further indicated the influence of N uptake and assimilation on the responses of plants to N forms and rates in C fixation or biomass production. In addition, Guo *et al*.^13,14^ found that N uptake under NH_4_^+^-fed was significantly higher than that under NO_3_^−^-fed during the dark period, whereas N uptake under NH_4_^+^-fed was significantly lower compared to that under NO_3_^−^-fed during the light period with a split root system in bean plants. Thus, we suggest that plant N uptake may be influenced not only by N forms, but also by the environmental condition, especially changes in light/dark conditions.

Furthermore, as an important osmoticum, K^+^ was significantly decreased in wheat plants under NH_4_^+^ nutrition (Fig. 2). Similar results have been reported for tobacco^17^, in which K^+^ uptake was inhibited under NH_4_^+^ nutrition, while K^+^ transport in the xylem and K^+^ translocation in the phloem was still higher than that in plants under NO_3_^−^ nutrition, although the process was improved more under high nutrient levels (6 mmol L^−1^ K and 15 mmol L^−1^ N) than under low levels (2.5 mmol L^−1^ K and 2 mmol L^−1^ N). Walch-Liu *et al*.^41^ found that the leaves of young, expanding and old tobacco plants under NH_4_^+^-fed showed 20%, 22% and 60% decreases in K concentrations, respectively, which indicated that K may mediate the effect of N forms on leaf morphogenesis. Most studies have revealed a negative effect of NH_4_^+^ nutrition on the osmotic regulation of leaves due to reduced K absorption^41,45,46^. These results suggest that this effect might be responsible for smaller leaf area and lower specific leaf weight as well as the reduction in root cell length and root morphological parameters frequently observed in rice plants under NH_4_^+^ nutrition^6,32,39^. Also, the K uptake by NH_4_^+^-fed vessels was significantly lower than that by NO_3_-fed vessels, while the K uptake rate was reduced by 466%^13^ and 1231%^14^, respectively, by the application of a split root system in bean plants. Similarly, in the present study, NH_4_^+^-fed resulted in a higher K^+^ uptake rate compared to that of NO_3_^−^-fed plants (Fig. 5). Thus, we suggest that plant K uptake may be influenced not only by the amount of available N in the environment, but also by the N forms.

### Interaction between N and K supply in wheat

The interaction between K and N, especially K and N forms (both NH_4_^+^ and NO_3_^−^) on plant growth and development, has become a focus of research^4,16,17,23,47,48^. Our studies in wheat showed that N forms affect plant growth and the uptake of N and K nutrients; however, the supply level of K also has a significant influence on the regulation of plant growth, photosynthesis and nutrient absorption, with a positive interaction identified between N and K. This is consistent with earlier results of studies in wheat^49^, rice^4^, tobacco^16,17^ and beans^13,14^, in which NH_4_^+^ nutrition not only strong inhibited K uptake, but also had a marked influence on the flow and partitioning within plants, resulting in reducted water uptake and *T*_r_, apparently due to reduced *g*_s_. This is consistent with the proposed theory that cycling of K in plants can act as an important signal for feedback control of nutrient uptake^50^. However, in this study, the growth variables, gas exchange parameters, and nutrient content of wheat plants were increased with increasing K supply, and the beneficial effects were observed under both NH_4_^+^ and NO_3_^−^ nutrition. These results further implied that optimal K management may alleviate NH_4_^+^ stress or toxicity and improve the nutritional function of NO_3_^−^ in wheat plants.

K is an essential macro-element of nutrition in plants and its uptake is strongly influenced by other elements, such as N. Elevating the K supply results in a significant reduction of NH_4_^+^ influx in rice plants^4^. Similarly, we observed that the growth of wheat plants under pure NH_4_^+^ nutrition was improved by K supply (Fig. 1), a phenomenon that demonstrates the influence of K supply on the tolerance of wheat to NH_4_^+^ stress or toxicity^49^. Based on the proposals of Szczerba *et al*.^4^ and Kong *et al*.^49^, we suggested that adequate K application is essential for plant growth, especially under conditions of high environmental NH_4_^+^ stress. The results of the present study also demonstrated that N and K interactions not only affect plant growth and nutrient uptake, but also significantly affect crop yield parameters, such as panicle biomass (Table 1). These results provide evidence that assimilate partitioning in plants can be improved by changes in K supply. Some studies suggest that the controversial effects of N forms and K supply on plant growth are related to the availability of photo-assimilates for production, transportation and distribution, further indicating that K plays an important role in yield formation^23,47,48^.

Although the plants did not show any visible symptoms of mineral nutrient deficiency under different N and K treatments, there were marked morphological differences between wheat plants, especially the shoots, for which the erecting and tilting degree were improved under high N and low K conditions under NO_3_^−^ nutrition compared with NH_4_^+^ nutrition (Fig. 1). Previous studies demonstrated that higher N rates reduced plant morphology traits, culm physical strength and lodging resistance in wheat^51^ and rice^52^, with every 2% increase in lodging resistance causing a 1% decrease in grain yield^53^. However, Zaman *et al*.^54^ found that optimizing K fertilizer improved stem strength and yield to alleviate the negative effects of higher N application. Kong *et al*.^49^ also reported that the additional provision of K^+^ considerably alleviated the negative effects of high NH_4_^+^, resulting in a 23% increase in culm mechanical strength and a 35% increase in the N remobilization efficiency in wheat plants. It has also been reported that the consequences of N metabolism^55^, N use efficiency^48^ and C-N balance^56^ are improved by K fertilizer application. Similarly, the results of the present study suggest that a positive synergistic interaction between K and N on assimilate production, nutrient uptake, yield formation and stress tolerance^47,57^. Thus, an understanding of the roles of the interaction between N (especially in NH_4_^+^) and K in the regulation of physiological and biochemical mechanical and yield formation is required to improve sustainable productivity in wheat plants.

In conclusion, wheat grown in anaerobic soils showed growth inhibition when the predominant form of nitrogen is NH_4_^+^ or a mixture of NH_4_^+^ and NO_3_^−^, and under conditions of limited K availability. NO_3_^−^ treatment increased root growth, N uptake and photosynthetic productivity in wheat plants in comparison with NH_4_^+^ treated plants, and the effects were enhanced by K supply, especially under high N conditions. Thus, our results indicate that increased K supply mediates nutrient balance between N and K uptake, and enhances NH_4_^+^ tolerance in wheat plants supplied with pure NH_4_^+^ nutrition. Our results provide the basis for the development of new nitrogen fertilizer utilization schemes for wetland wheat production.

## Materials and Methods

### Plant materials and growing conditions

A split-unit randomized complete block design, with N forms as the main unit and factorial combinations of N rates and K levels as subunits, was used in this study. The treatments were: two N forms (ammonium, as NH_4_^+^ [AN]; nitrate, as NO_3_^−^ [NN]), two N rates (2 and 10 mmol L^−1^), and three K levels (0.5, 5 and 15 mmol L^−1^ [K0.5, K5 and K15]) replicated three times. The ‘Yangmai 16’ hard red winter wheat (*Triticum aestivum* L.) was used as a model. Briefly, after germination on moist filter paper, wheat seeds were disinfected with 10% H_2_O_2_ for 30 min then transferred to a 2 mmol L^−1^ CaSO_4_ solution for germination at 25 ± 5 °C. When the seedlings had an average of 2.5 visible leaves (0.5 g fresh weight, 17 cm plant height), they were transplanted to 0.75 L (top diameter 9.5 cm × bottom diameter 6.5 cm × 16 cm high) plastic pots (two seedlings per pot) containing clean quartz sand and transferred into quarter-strength Hoagland’s nutrient solution (for composition, see below) with 1 cm depth in tray. Seedlings were grown in a greenhouse under a natural photoperiod. Four days later, the seedlings were transferred to half-strength nutrient solution. After an additional four days, the seedlings were treated with full-strength nutrient solution containing 12 different treatments (AN2K0.5, AN2K5, AN2K15, AN10K0.5, AN10K5, AN10K0.5, NN2K0.5, NN2K5, NN2K15, NN10K0.5, NN10K5, and NN10K15). The composition of the other nutrients in the solutions was as follows: macronutrients (mmol L^−1^): 2 or 10 N as (NH_4_)_2_SO_4_ or Ca(NO_3_)_2_, 0.5, 5 or 15 K as K_2_SO_4_ and KH_2_PO_4_, 1 P as KH_2_PO_4_, 5 Ca as CaCl_2_ or Ca(NO_3_)_2_, 2 Mg as MgSO_4_; micronutrients (μmol L^−1^): 100 Fe as Fe-EDTA, 9 Mn as MnCl_2_·4H_2_O, 0.7 Cu as CuSO_4_·5H_2_O, 0.7 Zn as ZnSO_4_·7H_2_O, 45 B as H_3_BO_3_, 1.7 Mo as (NH_4_)_6_Mo_7_O_24_·4H_2_O, and 100 Si as Na_2_SiO_3_·9H_2_O. The Ca content in the NH_4_^+^ nutrient solution was compensated for by the addition of CaCl_2_. A nitrification inhibitor (dicyandiamide, DCD) was added to prevent oxidation of NH_4_^+^ at a dose of 5% of total nitrogen. Nutrient solutions were changed every 4 d, after drip washing the sand surface with 0.5 L tap water, and the pH was monitored daily and maintained at 6.00 ± 0.05 by adding either 0.1 mmol L^−1^ NaOH or HCl until the final harvest. Each treatment group consisted of six plants in a completely randomized design to minimize edge effects.

### K^+^ uptake rate measurement

The examine the effect of N forms on K^+^ uptake rate, K^+^ influx of intact wheat plants was determined by the depletion of nutrient solution directly using a K^+^ concentration gradient method with N forms supplied under hydroponic solution conditions. Wheat seedlings grown in 2 mmol L^−1^ CaSO_4_ solution were pre-equilibrated for 2 h, then immersed in labelling solution between 8:30 to 16:30 (8 h). The solution was identical to the growth solution, except that it contained different K^+^ concentrations (0, 0.05, 0.1, 0.2, 0.4, 0.5, 1, 2, 4, 5, 10 mmol L^−1^) with the addition of 2 mmol L^−1^ AN or NN. Each treatment group consisted of three plants and was replicated three times in a completely randomized design. The seedlings were grown under greenhouse conditions (air temperature 30 °C; relative humidity 50%) under a photosynthetic photon flux density (PPFD) of 1000 μmol photons m^−2^ s^−1^ at the leaf level.

### Gas exchange measurements

At the heading stage, 60 days after treatments initiation, the light-saturated photosynthetic rates of newly expanded leaves (flag leaf) were measured simultaneously between 09:00 and 15:00 with an infrared gas analyzer (6400XT, Li-Cor, Lincoln, NE, USA). Leaf temperature during the measurements was maintained at 28 °C and a relative humidity of 50% under a PPFD of 1000 μmol photons m^−2^ s^−1^. Data were recorded after equilibration to a steady state.

### Relative chlorophyll content measurement

The one-dimensional (1 D) chlorophyll index of the labeled leaf segments was determined using a portable relative chlorophyll meter (SPAD-502, Minolta Camera, Osaka, Japan) to measure absorbance at 650 nm. The chlorophyll index was determined as the mean of six SPAD-502 readings from the same leaf at the heading stage.

### Root volume, dry weight and specific leaf weight measurements

Wheat plants were harvested and separated into root, stem (including sheath and culm), leaf and panicle sections. Sand was washed from the roots before the root volume was measured using the displacement method described by Sattelmacher *et al*.^58^. The dry weight was measured after all samples were oven-dried at 105 °C for 30 min and then at 70 °C to constant weight. The leaf area was determined using a photocopy of the leaf and calculated according to the paper area. The specific leaf weight was then calculated as the ratio of leaf weight to leaf area.

### N and K concentration and accumulation measurements

To determine the total concentrations of N and K in different organs, dried and ground samples were digested with H_2_SO_4_-H_2_O_2_ at 260–270 °C. N concentrations were measured using an Auto-analyzer 3 digital colorimeter (AA3, Bran + Luebbe, Hamburg, Germany) and K concentrations were measured by Flame Photometry (FP6400, Shanghai Precision Scientific Instrument, Shanghai, China). The measurements were validated using certified standard reference materials obtained from the Institute for Environmental Reference Materials of the Ministry of Environmental Protection (Beijing, China). The total accumulation of N and K was calculated from the sum of organ N and K content (element concentration × dry weight) at harvest.

### Statistical analysis

Samples were analyzed in triplicate and mean values were used in comparisons analysis. Variance analysis (ANOVA) was performed using the SAS 9.3 statistical software package (SAS Institute, Cary, NC, USA). Means were compared among treatments by the least significant difference (LSD) test with *P* < 0.05 considered to indicate statistical significance. Principal components analysis (PCA) was used to analyze the growth and physiological variations of NH_4_^+^, NO_3_^−^ and NH_4_^+^ plus NO_3_^−^ under treatment with different N rates and K supplies using SPSS Statistics 17.0 (IBM, Armonk, NY, USA) and plotted using SigmaPlot 12.5 (Systat Software Inc., San Jose, CA, USA).
